# High-Efficient Syndrome-Based LDPC Reconciliation for Quantum Key Distribution

**DOI:** 10.3390/e23111440

**Published:** 2021-10-31

**Authors:** Hao-Kun Mao, Yu-Cheng Qiao, Qiong Li

**Affiliations:** 1 Department of Computer Science and Technology, Harbin Institute of Technology, Harbin 150080, China; 14B303003@hit.edu.cn; 2 Guangxi Key Lab Cryptography & Information Security, Guilin University of Electronic Technology, Guilin 541004, China; glqyc251@guet.edu.cn

**Keywords:** quantum key distribution, blind reconciliation, low density parity check, syndrome

## Abstract

Quantum key distribution (QKD) is a promising technique to share unconditionally secure keys between remote parties. As an essential part of a practical QKD system, reconciliation is responsible for correcting the errors due to the quantum channel noise by exchanging information through a public classical channel. In the present work, we propose a novel syndrome-based low-density parity-check (LDPC) reconciliation protocol to reduce the information leakage of reconciliation by fully utilizing the syndrome information that was previously wasted. Both theoretical analysis and simulation results show that our protocol can evidently reduce the information leakage as well as the number of communication rounds.

## 1. Introduction

Quantum key distribution (QKD) is a promising technique to share secure keys via insecure quantum and classical channels between two remote parties, usually called Alice and Bob [1]. Unlike conventional cryptography, QKD is based on quantum mechanical principles and is guaranteed to be secure without assumptions of adversary’s (i.e., Eve’s) computing power or technological capability [2,3]. For this reason, QKD has been developing rapidly in both theory and experiment during the last few decades [4,5,6,7,8,9,10,11,12,13]. In fact, QKD has become one of the most mature branches of quantum information technology [14,15,16]. The workflow of a QKD system can be divided into two main phases [2], namely the quantum phase and the post-processing phase. In the quantum phase, the QKD system deals with state preparation, transmission and detection of quantum signals, and then only retains the keys with matched bases (i.e., sifted keys). However, due to non-ideal factors in the actual process, including channel noise, eavesdropper interference, etc., there are errors in the sifted keys, and some information may be revealed. To correct the errors and remove the Eve’s information about the sifted keys, the two parties sequentially perform two necessary steps in the post-processing phase, namely, the information reconciliation (IR) step and the privacy amplification (PA) step. It should be noted that PA usually performs on a number of IR frames (hereafter referred to simply as frame) to deal with the finite-size-effect [2]. After post-processing, the identical and information-theoretically secure keys are finally shared between Alice and Bob. In this paper, we focus on the IR step, which is obviously a vital step of QKD [17], for the errors introduced in the quantum phase must be eliminated to obtain a correct and secure key sequence available in cryptography.

Many IR protocols have been proposed aiming at minimizing the information leakage of IR, i.e., improving the IR efficiency. The terms of IR protocols and IR efficiency are hereafter referred to simply as protocols and efficiency, respectively. Among all these protocols, the low-density parity-check (LDPC) code based protocols [18,19,20,21,22] have received much attention in recent years for its advantages of inherent good parallelism, high efficiency and less communication consumption. LDPC code is a linear block code given by a parity-check matrix $H_{m\times n}$ with code rate of R=1−m/n, where *m* and *n* represent the lengths of syndrome $S_{m\times 1}$ and frame $F_{n\times 1}$, respectively. LDPC codes were first applied in discrete-variable (DV) QKD systems in [23]. In early QKD systems, the LDPC codes were used in a straightforward way. For an estimated quantum bit error rate (QBER), a suitable LDPC code was first chosen from a code-set or specially designed [24]. Then, the syndrome *S* was generated as S=H·F and all the *m* syndrome bits of *S* were transmitted to the other party for LDPC decoding. In this way, a high efficiency could be achieved only when the actual QBER of the frame fluctuated in a narrow region around the estimated QBER. Specifically, for a lower QBER, the efficiency would decrease due to excessive information leakage, and for a higher QBER, the efficiency would also decrease due to an increase in the frame error rate (FER), i.e., the rate of frames that cannot be corrected. However, for a QKD system operating in a practical condition, its QBER might vary significantly in consecutive frames [17,25]. To produce high efficiencies in a range of possible QBERs, many highly efficient large frame-length LDPC codes that were difficult to design, as well as a highly accurate estimation of QBER that might be unpredictable in a practical QKD system, were needed.

To overcome such shortcomings, the rate-adaptive protocol was proposed [20]. In this protocol, the two parties agreed on some bits of the frame to be treated as modulated bits, including punctured bits (i.e., true random bits that were generated in both parties independently) and shortened bits (i.e., published bits that were known with absolute certainty to both parties as well as Eve). By utilizing modulated bits, *R* could be adjusted to adapt to the QBER fluctuations, thus higher efficiencies within a wider QBER range were achieved with only one LDPC code. However, this protocol still needed a priori estimation of the QBER and large frame-length LDPC codes. To address this issue, the blind protocol was proposed [21]. On the basis of the rate-adaptive protocol, the blind protocol introduced additional interactivity into the IR process. Specifically, the protocol required two parties to assume the maximum number of punctured bits in the first communication round. Once the decoding failed, Alice published a small fixed number of punctured bits, which would help Bob to resume the decoding. In this way, a blind protocol could work without a priori QBER and enhance the efficiency under the circumstance of the fluctuated QBER in practice. In addition, this protocol could achieve high efficiencies within a wider QBER range even with frame-length of 2 kb, which made it suitable for hardware implementations. Later in [26], an increasing number instated of a fixed number of punctured bits were revealed in each additional communication round. With the help of this optimization, Bob could receive increasing help from Alice in the later communication round, thereby the convergence of IR was accelerated. To further improve the efficiency while reducing the interactivity, the symmetric-blind reconciliation protocol was proposed [22] via introducing symmetry in operations of two parties and consideration on unsuccessful decoding results. Collectively, these LDPC-based protocols have their own application scope. For long frame-length, the rate-adaptive protocol is a good solution, while for short frame-length, the blind or symmetric-blind protocol may be a better choice.

Although LDPC-based protocols have been well studied, we find out that there remains much room for performance improvement in a practical QKD system. We notice that an LDPC syndrome *S* of length *m* is considered to leak *m*-bit information to Eve and this m-bit information will be discarded in the PA step. However, the actual error rate between Alice’s and Bob’s syndromes is usually not equal to, but less than 50% in a practical IR implementation. This observation indicates that the actual amount of information obtained by Eve is less than *m*-bit, and some syndrome information has been wasted. If this wasted syndrome information can be used in LDPC decoding, the efficiency can be further improved. In light of this, we propose a novel LDPC-based protocol to fully utilize this wasted syndrome information. In our protocol, we focus on a set of frames rather than a single frame. By replacing some punctured bits from random bits with syndrome bits, the previous wasted syndrome information can be taken as an advantage in our protocol. The efficiency of our protocol is proved to be better than previous LDPC protocols. In addition, our protocol is easy to implement. Simulation results show that our protocol achieves better efficiency and less communication consumption than the comparative protocols.

The rest of this paper is organized as follows. Section 2 gives a brief description of some basic concepts of IR and the related LDPC-based protocols. In Section 3, the details of our protocol are presented. The efficiency and effectiveness of our protocol in a practical QKD system are also analyzed. The simulations results compared to the rate-adaptive and symmetric-blind protocols are reported and discussed in Section 4. Some conclusions are drawn in the last section.

## 2. LDPC-Based IR Protocols

In a DV-QKD system, the discrepancies between Alice’s and Bob’s frames can be assumed to be the transmission result over a binary symmetric channel (BSC) [27] with crossover probability *q*, which is usually referred to as QBER. Therefore, for a frame of length *n*, the theoretical lower limit of the revealed information for successful reconciliation can be calculated by nh(q), where h(q)=−qlog(q)−(1−q)log(1−q). Knowing that R=1−m/n and all *m* syndrome bits are leaked to Eve in the previous LDPC protocols, an important performance metric *f* for IR is given as

(1) $$f=\frac{m}{nh(q)}=\frac{1-R}{h(q)}.$$

Obviously, *f* is always greater than 1, and the smaller *f* is, the better IR performs.

### 2.1. Rate-Adaptive Protocol

For a LDPC code with fixed *R*, we know from Equation (1) that *f* only remains high within a narrow range around *q*. However, *q* might vary significantly in two consecutive frames, especially for a real QKD setup operating in an urban environment [17]. To this end, the rate-adaptive protocol was proposed [20], in which *R* could be adjusted to the desired efficiency $f_{desire}$ by padding extra *p* punctured bits and *s* shortened bits, thus the frame to be reconciled consists of *p* punctured bits, *s* shortened bits and (n−p−s) sifted keys. Since the shortened (punctured) bits decreased (increased), the code rate, a proper *R* could be achieved by balancing the values of *p* and *s*. Let d=p+s represent the initial number of modulated bits, the modulated rate *R* was then calculated as

(2) $$R=\frac{n-m-s}{n-d},$$

and the range of achievable code rates could be obviously derived as

(3) $$R_{min}=\frac{n-m-d}{n-d}\leq R\leq \frac{n-m}{n-d}=R_{max}.$$

In this way, the rate-adaptive protocol could cover a wide range of QBER by using only one LDPC code. Based on Equations (1) and (2), the efficiency *f* for the rate-adaptive protocol was obtained as

(4) $$f=\frac{m-p}{\left(n-d)h(q\right)},$$

where *q* was the actual QBER. Accordingly, to achieve a desired efficiency $f_{desire}$ with the estimated QBER $q_{est}$, we could derive the optimal values of *s* and *p* as

(5) $$\begin{array}{cc} s & =\left\lceil \left(n-m\right)-\left(n-d\right)\left(1-f_{desire}h\left(q_{est}\right)\right)\right\rceil ,\\ p & =d-s. \end{array}$$

### 2.2. Blind Protocols

Instead of calculating the values of *s* and *p* at the beginning of the protocol, the blind protocol [21] started without error estimation and all *d* bits were initially regarded as punctured bits. In case of a decoding failure, the decoding process would resume again by revealing some punctured bits in the next round, i.e., these punctured bits were transformed into shortened bits. In this way, a high *f* was achieved even with a short frame length. According to Equation (4), *f* for blind protocol could be calculated as

(6) $$f=\frac{m-p+\Delta }{\left(n-d)h(q\right)},$$

where Δ was the total number of the shortened bits in the additional communication rounds. Actually, *d* modulated bits could also contain *s* initial shortened bits, s.t. d=p+s. Thus, Equation (6) held for the rate-adaptive protocol as well.

To further improve the efficiency, the symmetric-blind protocol was proposed by introducing symmetry in operations of two parties and consideration on unsuccessful decoding results [22]. Though *f* could also be calculated by Equation (6), this protocol used a quite different approach to obtain the desired efficiency $f_{desire}$, that is, only shortened or punctured bits were used. For each code among the code set, the number of shortened or punctured bits was calculated as follows. Let $f=m/\left[nh(q)\right]$, for $f>f_{desire}$,

(7) $$\begin{array}{cc} p & =\left\lfloor m-nh\left(q_{est}\right)f_{desire}\right\rfloor /\left(1-h\left(q_{est}\right)f_{desire}\right),\\ s & =0, \end{array}$$

while for $f<f_{desire}$,

(8) $$\begin{array}{cc} s & =\left\lceil n-m/h\left(q_{est}\right)f_{desire}\right\rceil ,\\ p & =0. \end{array}$$

The LDPC code that maximized the number of sifted keys was then chosen. In addition, in case of decoding failure, the shortened bits were not chosen randomly from the punctured bits as in the blind protocol, but from the bits with the minimal values of log-likelihood ratio (LLR) magnitude, which contributed to the decoding process. Since the shortened bits were not only selected from the punctured bits, IR could continue even in the absence of punctured bits. In this way, the convergence was formally guaranteed by the fact that in the worst-case scenario, all the bits of the frame were revealed [22].

## 3. Syndrome-Based LDPC Reconciliation

### 3.1. The Main Idea of Our Protocol

**Figure 1:**
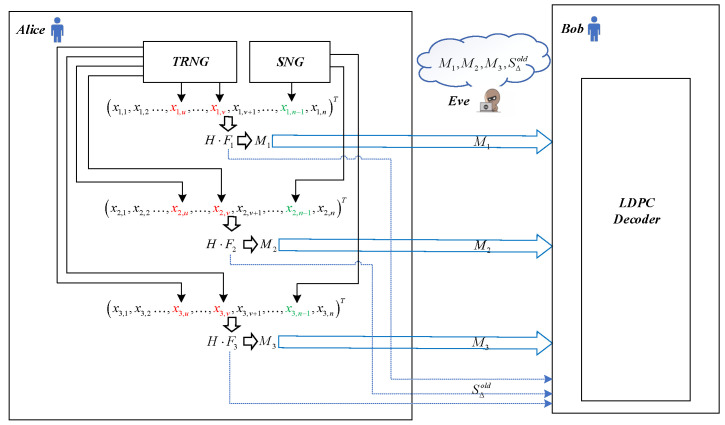
Simplified schematic workflow of the previous LDPC protocols. The punctured bits and shortened bits are labeled in red and green, respectively. The blue dashed lines indicate the additional communications.

**Figure 2:**
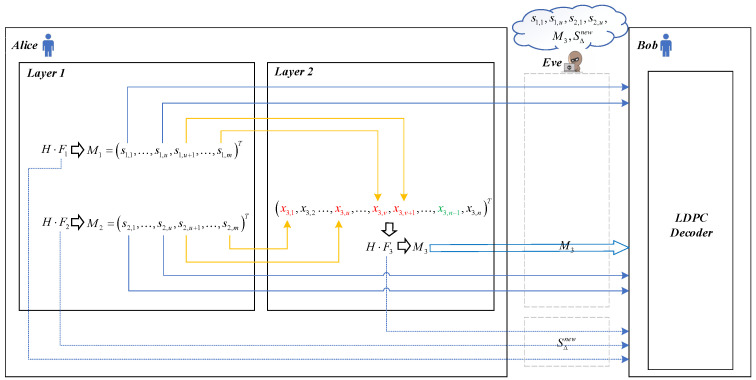
Simplified schematic workflow of our protocol. The orange lines indicate that some punctured bits of Layer 2 are replaced by some syndrome bits generated by frames in Layer 1. Other descriptions are the same as in Figure 1.

In this part, we elaborate upon the main idea of our protocol and analyze its improvement in efficiency by contrasting the workflow of the previous LDPC protocols (schematically shown in Figure 1) with our protocol (schematically shown in Figure 2). We first assume that there are *w* frames to be reconciled and consider these frames as a whole when calculating the efficiency. Since Alice and Bob perform the same operation when constructing the frames, without loss of generality, we mainly focus on the operations at Alice’s side. In addition, in both Figure 1 and Figure 2, $F_{i}$ and $M_{i}$ represent the ith frame of length *n* and the ith syndrome of length *m*, respectively.

In the previous LDPC protocols, each $F_{i}$ is first constructed by padding *p* punctured bits, *s* shortened bits and (n−p−s) sifted keys. As shown in Figure 1, the punctured bits are generated by a true random number generator (TRNG) and thus unknown to any other party. In contrast, the shortened bits are generated by a shared number generator (SNG) and exactly known by all parties, including Bob and Eve. Note that the SNG can be a pseudo random number generator (PRNG) with a shared initial seed. After construction, each $M_{i}$ is generated by $H\cdot F_{i}$ and then transmitted directly through the classical channel to Bob for LDPC decoding. After decoding, Bob will send an acknowledgement back to Alice to indicate whether the decoding succeeds. If successful, the frame will be outputted to the subsequent PA step. Otherwise, some frame bits will be published and transformed into shortened bits. With these new published bits (i.e., additional shortened bits), Bob will resume LDPC decoding. For all the *w* frames, after several additional communication rounds, the decoding process will eventually succeed with the help of the additional shortened bit sequence $S_{\Delta }^{old}$. According to Equation (6), the efficiency $f^{old}$ of a previous protocol can be calculated as

(9) $$f^{old}=\frac{m^{old}-p^{old}+\left|S_{\Delta }^{old}\right|}{\left(n^{old}-p^{old}-s^{old}\right)h\left(q\right)},$$

where $n^{old}$, $m^{old}$, $p^{old}$, $s^{old}$ represent the total number of input frame bits, syndrome bits, initial punctured bits, and initial shortened bits, respectively.

For our protocol, the main idea is to replace some punctured bits from random bits with syndrome bits to fully utilize the previously wasted syndrome information. To this end, we divide the frames to be reconciled into two layers $L_{i}\left(i=1,2\right)$ as shown in Figure 2. The frames and syndromes of $L_{1}$ are first constructed and generated as in the previous LDPC protocols, respectively. Then, some syndrome bits of these syndromes are used to initialize the punctured bits of the frames in $L_{2}$, and the others are transmitted together with the syndromes of $L_{2}$ to Bob. Let *T* represent the set of punctured bits in $L_{2}$ that are initialized by the syndrome bits from $L_{1}$ (e.g., $T=\left\{x_{3,1},x_{3,u},x_{3,v},x_{3,v+1}\right\}$ in Figure 2), the efficiency of our protocol can be calculated according to Equation (6) as

(10) $$f^{new}=\frac{m^{new}-p^{new}+\left|S_{\Delta }^{new}\right|}{\left(n^{new}-p^{new}-s^{new}-\left|T\right|\right)h\left(q\right)},$$

where $n^{new}$, $m^{new}$, $p^{new}$, $s^{new}$, $S_{\Delta }^{new}$ represent the total number of input frame bits, syndrome bits actually published, initial punctured bits, initial shortened bits, and the additional shortened bit sequence, respectively.

We next analyze the relationship between $f^{new}$ and $f^{old}$. Since the total input size of reconciliation remains constant, we have $n^{new}=n^{old}$. According to the definitions of $m^{new}$, $m^{old}$ and *T*, we have $m^{old}=m^{new}+\left|T\right|$. As discussed in Section 1, the actual error rate of the syndromes in $L_{1}$ is usually less than 50%. Benefiting from this fact, when our protocol is applied, the frames in $L_{2}$ can be decoded successfully even with more modulated bits. Therefore, we have $p^{old}+s^{old}<p^{new}+s^{new}+\left|T\right|$. In addition, because the difficulty of LDPC decoding the frames in $L_{2}$ also decreases with the help of lower error rate of punctured bits, fewer shortened bits are needed, that is, $s^{new}+\left|S_{\Delta }^{new}\right|<s^{old}+\left|S_{\Delta }^{old}\right|$. In summary, according to Equations (9) and (10), we have,

(11) $$\begin{array}{cc} f^{new} & =\frac{\left(m^{old}-\left|T\right|\right)-p^{new}+\left|S_{\Delta }^{new}\right|}{\left(n^{old}-p^{new}-s^{new}-\left|T\right|\right)h\left(q\right)},\\ \; & =\frac{m^{old}-\left(p^{new}+s^{new}+\left|T\right|\right)+\left(s^{new}+\left|S_{\Delta }^{new}\right|\right)}{\left[n^{old}-\left(p^{new}+s^{new}+\left|T\right|\right)\right]h\left(q\right)},\\ \; & <\frac{m^{old}-\left(p^{old}+s^{old}\right)+\left(s^{new}+\left|S_{\Delta }^{new}\right|\right)}{\left(n^{old}-p^{old}-s^{old}\right)h\left(q\right)},\\ \; & <\frac{m^{old}-\left(p^{old}+s^{old}\right)+\left(s^{old}+\left|S_{\Delta }^{old}\right|\right)}{\left(n^{old}-p^{old}-s^{old}\right)h\left(q\right)},\\ \; & =f^{old}, \end{array}$$

which indicates that our protocol theoretically performs better than the previous LDPC protocols in terms of efficiency.

Let $M_{1}$ represent the set containing all syndrome bits of $L_{1}$. It should be noted that when applying the puncturing technique, not all syndrome bits in $M_{1}$ can be used to initialize the bits of *T*. Let $M_{p}$ and $M_{k}$ represent the sets of syndrome bits in $M_{1}$ that are relevant and irrelevant to the punctured bits, respectively, s.t. $M_{p}\cup M_{k}=M_{1}$. Obviously, the error rates of $M_{p}$ and $M_{k}$ are equal to and less than 50%, respectively. Since our protocol is effective only when the punctured bits are initialized by the syndrome bits of $M_{k}$, we only focus on $M_{k}$ in the rest of the discussion. On the basis of our main idea, we now proceed to describe the workflow of our protocol.

### 3.2. Description of Our Protocol

The Specific Procedure of Our Protocol is as Follows.

**Step 0: Initialization**. Alice and Bob divide the frames to be reconciled into two layers. The frames in $L_{1}$ are first initialized according to the protocol based on, such as rate-adaptive, blind or symmetric-blind. Then, the two parties generate the syndromes of $L_{1}$ but do not publish them.

**Step 1: Syndrome puncturing**. Alice and Bob sequentially initialize the punctured bits *T* by the syndrome bits from $M_{k}$. If $\left|M_{k}\right|>\left|T\right|$ (i.e., $\left|T\right|$ is insufficient to contain all syndrome bits of $M_{k}$), the remaining $\left(\left|M_{k}\right|-\left|T\right|\right)$ syndrome bits are then transmitted to the other party. Otherwise, the remaining $\left(\left|T\right|-\left|M_{k}\right|\right)$ bits are initialized by true random bits. In this situation, the length $\left|T\right|$ decreases to $\left|M_{k}\right|$. Then, the syndromes of $L_{2}$ are generated and transmitted to the other party through the classical channel.

**Step 2: Layered decoding**. The interactive decoding process of $L_{2}$ starts first. Once the frames in $L_{2}$ are corrected, $L_{1}$ starts interactive decoding next. Note that in the LLR initialization step, the LLRs for bits of *T* are initialized according to the theoretical error rate of $M_{k}$, and the LLRs for other bits are initialized as in the protocol it is based on.

We note that the principal difference between our protocol and the previous protocols is that our protocol adds an additional step “Step 1” and needs to control the decoding processes between layers in “Step 2”. Because these additional costs are negligible, we believe our protocol is low cost and easy to implement. We can also see from “Step 0” that our protocol is compatible with nearly all commonly used LDPC protocols.

The performance improvement of our protocol is attributed to its full utilization of the wasted syndrome information. It is important to note that our protocol performs well under three conditions as described below. First, our protocol requires several frames to be reconciled together. Second, the error rate of syndrome bits of $M_{k}$ is needed to be less than 50%. Third, $\left|T\right|$ needs to be large enough. If the above conditions are not satisfied, the performance improvement will degrade. For this reason, we next analyze whether the above conditions are easy to meet in a practical QKD system, that is, the effectiveness of our protocol.

### 3.3. Effectiveness Analysis of Our Protocol

#### 3.3.1. Number of IR Frames

We note that the typical frame lengths for blind and rate-adaptive protocols are 2 [21,22] and 100 kb [18,19,20], respectively. We notice that the frame length of reconciliation is usually not long enough in a practical QKD system to facilitate the LDPC code design and practical IR implementation. However, the input size of PA is usually between $10^{6}$ and $10^{8}$ to deal with the finite size effect. Thus, PA usually requires hundreds of IR frames, which satisfies the assumption of our protocol.

#### 3.3.2. Error Rate of $M_{k}$

In addition, let ε represent the error rate of $M_{k}$. According to the information theory, to reconcile with a number of transmitted syndrome bits close to the theoretical limit, each syndrome bit should contain (almost) one bit of information (that is, ε is close to 50%), which is most beneficial to the efficiency. However, to increase the convergence speed of LDPC decoding and to decrease the FER, ε in practice is usually not equal to, but less than 50%, satisfying the requirements of our protocol.

**Figure 3:**
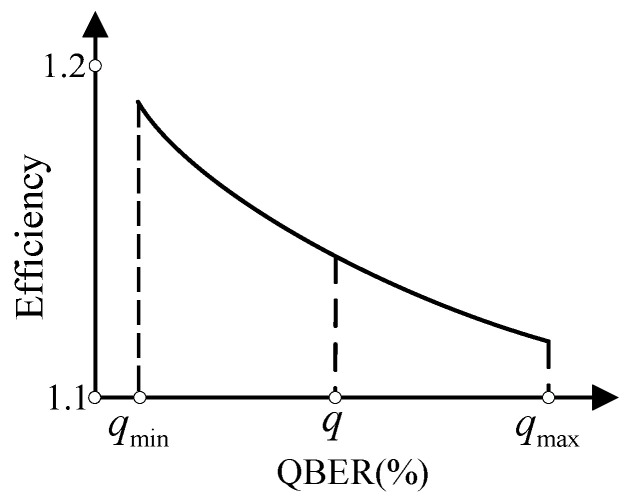
A typical efficiency curve for an LDPC code when puncturing and shortening techniques are applied.

Even when an LDPC code is designed for a desired QBER $q_{max}$ to make the corresponding ε equal to 50%, there still exists some QBERs with ε<50%. This is because an LDPC code is generally designed according to $q_{max}$ and is responsible for the QBERs within $\left[q_{min},q_{max}\right]$ when puncturing and shortening techniques are applied. As *q* decreases from $q_{max}$ to $q_{min}$, the ε decreases from 50% rapidly. In other words, the farther *q* is from $q_{max}$, the greater performance enhancement our protocol provides in terms of the efficiency. Similarly, as observed from Figure 3, the efficiency worsens when *q* strays away from $q_{max}$.

In addition to the above-mentioned scenario in which puncturing and shortening techniques are applied, our protocol is also well suitable for the scenario in which the standard LDPC codes are applied. As we know, it is widely accepted in the quantum cryptography community that designing a highly efficient LDPC code is really a challenging task. For this reason, the off-the-shelf standard LDPC codes have been widely used in industrial QKD systems [22,28]. The standard LDPC codes, such as IEEE 802.11n [29] and IEEE 802.16e [30], have low row-weights, which make them fit well to our protocol. For instance, to reconcile frames with QBER of 1.0%, we choose the most appropriate code rate R=5/6 with the frame length n=1944. This code has 243 and 81 parity-check rows with row-weights equal to 20 and 19, respectively. The theoretical value of ε is approximately 16.4%, which is far less than 50%. This suggests that our protocol can achieve high performance improvement when the standard LDPC codes with low row-weights are applied.

Next, we analyze whether $\left|T\right|$ can meet the requirements of our approach as mentioned above.

#### 3.3.3. Size of *T*

From “Step 0” of our protocol, we know that our protocol is based on the previous LDPC-based protocols. The most commonly used LDPC-based protocols are rate-adaptive, blind and symmetric-blind protocols. Based on each protocol, we analyze the corresponding $\left|T\right|$ in our protocol. The typical number of modulated bits for these protocols is shown in Table 1. From Table 1, we know that the blind protocol best fits the requirements of our protocol among these three protocols. This is because in the blind protocol, the typical proportion of *p* to *n* is 10%, which provides enough available punctured bits for *T*. For the rate-adaptive protocol, the situation is similar to that in blind protocol when *q* is around $q_{min}$. However, as *q* increases to $q_{max}$, the corresponding *p* decreases, resulting in a decrease in $\left|T\right|$. $\left|T\right|$ is similar in rate-adaptive and symmetric-blind protocol, except for one main difference, that is, for some QBERs in the symmetric blind protocol, only shortened bits are used. In this situation, there are no available punctured bits and our protocol may be ineffective. However, we can choose another LDPC code with p≠0 or design a proper LDPC code to avoid such QBER region in which our protocol performs worse, and in turn, make better use of our protocol.

## 4. Simulations and Discussions

In our simulation, we apply rate-adaptive and symmetric-blind protocols in “Step 0” of our protocol, respectively. The performance of our protocol is also compared with these two protocols. We design a set of QC-LDPC codes with n=100 kb for the rate-adaptive protocol. The variable node degree distributions and the masking matrices are optimized by using the density evolution (DE) algorithm [24] and standard PEG algorithm [31], respectively. For the symmetric-blind protocol, a set of standard LDPC codes with n=1944 are used as in [22]. Because the current QKD systems are typically running under q<3% and the secure key rate is rather low in the case of q>5%, we simulate *q* from 1% to 5%, which most current DV-QKD systems focus on. In terms of LDPC decoding, we use our previous reported simplified LDPC decoding algorithm and the syndrome-based early termination strategy [19].

**Figure 4:**
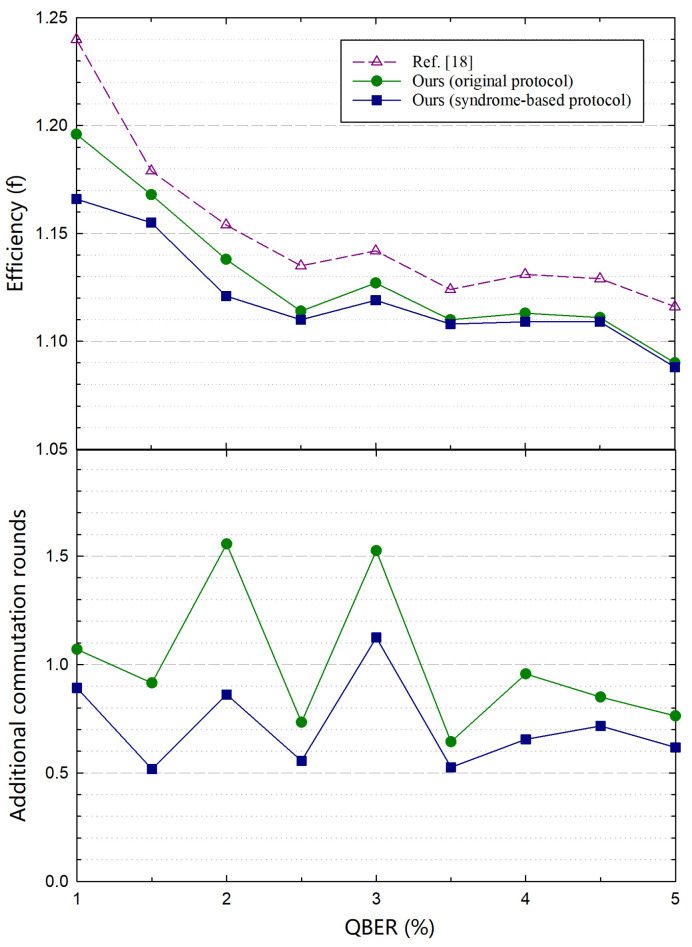
Comparison of the efficiencies (upper panel) and additional communication rounds (lower panel) with the rate-adaptive protocol. The short-dashed line with empty symbols stands for the simulation results from Reference [18] and is regarded as a benchmark. The solid lines with symbol types of squares and circles stand for our simulation results with the original protocol and our proposed protocol, respectively. Note that the communication rounds of Reference [18] are not plotted in our figure since these results were not given.

From the comparison results in Figure 4, we can see that our simulation with the rate-adaptive protocol performs better than the simulation results from [18] in terms of efficiency. The main reason is that we use more optimal LDPC codes and introduce interactivity into reconciliation. On this basis, by using our protocol based on the rate-adaptive protocol, we further improved efficiency while reducing the number of additional communication rounds. The performance improvement was particularly noticeable for low QBERs where the previous work did not perform well enough. In addition, we notice that the efficiencies of an LDPC code change consistently with our previous analysis in Section 3.3. For instance, for $q\in \left[2.0\%,2.5\%\right]$, the efficiency improvement decreases when *q* gradually increases to 2.5%. However, these results do not hamper the effectiveness of our protocol. This is because the efficiency is more determined by the intrinsic performance of an LDPC code than the adaptivity of an LDPC protocol when *q* approaches to the QBER according to which the LDPC code is designed.

**Figure 5:**
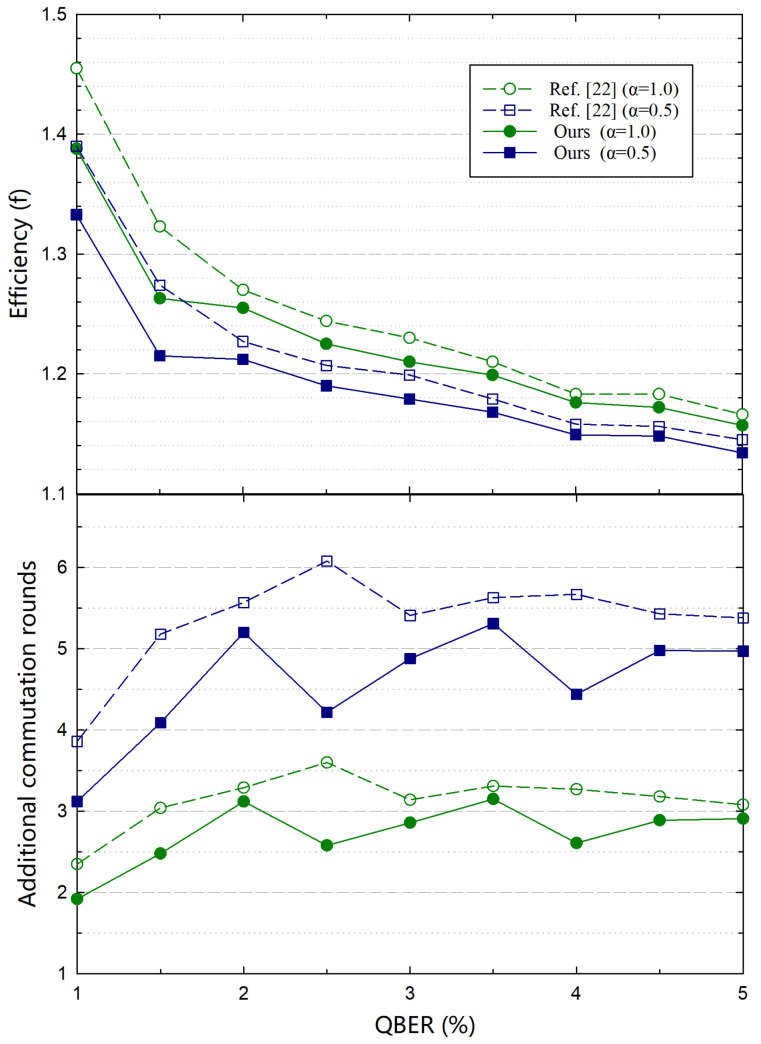
Comparison of the efficiencies (**upper panel**) and additional communication rounds (**lower panel**) with the symmetric-blind protocol. α is an auxiliary parameter defined in [22]. For a fixed LDPC code rate, a larger α means a larger shortening step. The lines with types of circle and square represent α=1.0 and α=0.5, respectively.

For comparison with symmetric-blind protocols, we apply a set of standard LDPC codes from the IEEE 802.11n [29]. From Figure 5, we can see that our protocol based on the symmetric-blind protocol performs better than the comparative schemes [22]. Compared with the above simulation results of our protocol based on the rate-adaptive protocol, our protocol gains a better efficiency improvement based on the symmetric-blind protocol, since the LDPC codes with low row-weights which facilitate our protocol are applied in our simulations. Encouraged by the outlined performance with the standard LDPC codes, our protocol can be applied in more practical QKD systems. Aside from the advantage of avoiding the challenging task of designing a highly efficient LDPC code, there are some other strengths when the standard LDPC codes are applied. For instance, the chip-based decoder with standard LDPC codes have been widely used in the field of classical communication. If these mature products can be applied in QKD systems, the overall system costs for QKD systems (e.g., volume, economic cost, power consumption, etc.) can be further reduced. Note that for a symmetric-blind protocol, there may exist only shortened bits for some QBERs. In this case, our protocol can not be applied directly. To overcome such issue, we may add some limitations that only the LDPC codes with punctured bits can be chosen. Since our protocol can significantly improve the performance of the original protocols, in most cases, we can achieve a better performance.

## 5. Conclusions

In this paper, we propose a novel syndrome-based LDPC protocol with high efficiency and low communication consumption. This protocol focuses on a set of frames rather than a single frame. By applying the novel syndrome-puncturing technique, the previous wasted syndrome information can be taken advantage of in our protocol. Furthermore, our protocol is easy to implement and compatible with nearly all commonly used LDPC reconciliation protocols. Simulation results show that our protocol not only improves the efficiency but also decreases the mean number of communication rounds. Note that our protocol is particularly suitable for low QBERs, which most DV-QKD systems focus on.

Furthermore, our protocol may have implications for other forward-error-correction code-based reconciliation protocols. If we can fully utilize the wasted information leakage caused by the transmitted bits with an error rate less than 50%, the efficiency can be further improved.

## Figures and Tables

**Table 1:** Comparison of the typical number of modulated bits in LDPC-based protocols. The definitions of *p*, *s*, *n* are the same as in Section 2.

LDPC-Based Protocol	Typical Number of Initial Punctured and Shortened Bits
Blind	p=n·10%, s=0
Rate-adaptive	p+s=n·10%
Symmetric-blind	p>0, s=0 **or** p=0, s>0

## Data Availability

Not Applicable.

## References

[1] Bennett C.H., Brassard G. (2014). Quantum cryptography: Public key distribution and coin tossing. Theor. Comput. Sci..

[2] Yuan Z., Plews A., Takahashi R., Doi K., Tam W., Sharpe A., Dixon A., Lavelle E., Dynes J., Murakami A. (2018). 10-Mb/s quantum key distribution. J. Light. Technol..

[3] Abd EL-Latif A.A., Abd-El-Atty B., Venegas-Andraca S.E., Mazurczyk W. (2019). Efficient quantum-based security protocols for information sharing and data protection in 5G networks. Future Gener. Comput. Syst..

[4] Lucamarini M., Yuan Z.L., Dynes J.F., Shields A.J. (2018). Overcoming the rate–distance limit of quantum key distribution without quantum repeaters. Nature.

[5] Wang X.B., Yu Z.W., Hu X.L. (2018). Twin-field quantum key distribution with large misalignment error. Phys. Rev. A.

[6] Fan-Yuan G.J., Wang Z.H., Wang S., Yin Z.Q., Chen W., He D.Y., Guo G.C., Han Z.F. (2021). Optimizing Decoy-State Protocols for Practical Quantum Key Distribution Systems. Adv. Quantum Technol..

[7] Ma X., Zeng P., Zhou H. (2018). Phase-matching quantum key distribution. Phys. Rev. X.

[8] Zhou Y.H., Yu Z.W., Wang X.B. (2016). Making the decoy-state measurement-device-independent quantum key distribution practically useful. Phys. Rev. A.

[9] Wang S., He D.Y., Yin Z.Q., Lu F.Y., Cui C.H., Chen W., Zhou Z., Guo G.C., Han Z.F. (2019). Beating the fundamental rate-distance limit in a proof-of-principle quantum key distribution system. Phys. Rev. X.

[10] Chen J.P., Zhang C., Liu Y., Jiang C., Zhang W., Hu X.L., Guan J.Y., Yu Z.W., Xu H., Lin J. (2020). Sending-or-not-sending with independent lasers: Secure twin-field quantum key distribution over 509 km. Phys. Rev. Lett..

[11] Yu Z.W., Hu X.L., Jiang C., Xu H., Wang X.B. (2019). Sending-or-not-sending twin-field quantum key distribution in practice. Sci. Rep..

[12] Cao Y., Li Y.H., Yang K.X., Jiang Y.F., Li S.L., Hu X.L., Abulizi M., Li C.L., Zhang W., Sun Q.C. (2020). Long-distance free-space measurement-device-independent quantum key distribution. Phys. Rev. Lett..

[13] Zhang Y., Chen Z., Pirandola S., Wang X., Zhou C., Chu B., Zhao Y., Xu B., Yu S., Guo H. (2020). Long-distance continuous-variable quantum key distribution over 202.81 km of fiber. Phys. Rev. Lett..

[14] Li Y., Zhang X., Li Y., Xu B., Ma L., Yang J., Huang W. (2020). High-throughput GPU layered decoder of quasi-cyclic multi-edge type low density parity check codes in continuous-variable quantum key distribution systems. Sci. Rep..

[15] Abd El-Latif A.A., Abd-El-Atty B., Mazurczyk W., Fung C., Venegas-Andraca S.E. (2020). Secure data encryption based on quantum walks for 5G Internet of Things scenario. IEEE Trans. Netw. Serv. Manag..

[16] Abd el Latif A.A., Abd-el Atty B., Amin M., Iliyasu A.M. (2020). Quantum-inspired cascaded discrete-time quantum walks with induced chaotic dynamics and cryptographic applications. Sci. Rep..

[17] Kiktenko E.O., Malyshev A.O., Fedorov A.K. (2020). Blind information reconciliation with polar codes for quantum key distribution. IEEE Commun. Lett..

[18] Dixon A., Sato H. (2014). High speed and adaptable error correction for megabit/s rate quantum key distribution. Sci. Rep..

[19] Mao H., Li Q., Han Q., Guo H. (2019). High-throughput and low-cost LDPC reconciliation for quantum key distribution. Quantum Inf. Process..

[20] Elkouss D., Martinez-Mateo J., Martin V. (2011). Information reconciliation for quantum key distribution. Quantum Inf. Comput..

[21] Martinez-Mateo J., Elkouss D., Martin V. (2012). Blind reconciliation. Quantum Inf. Comput..

[22] Kiktenko E.O., Trushechkin A.S., Lim C.C.W., Kurochkin Y.V., Fedorov A.K. (2017). Symmetric blind information reconciliation for quantum key distribution. Phys. Rev. Appl..

[23] Elliott C., Colvin A., Pearson D., Pikalo O., Schlafer J., Yeh H. (2005). Current status of the DARPA quantum network. Quantum Information and Computation III.

[24] Elkouss D., Leverrier A., Alléaume R., Boutros J.J. Efficient reconciliation protocol for discrete-variable quantum key distribution. Proceedings of the 2009 IEEE International Symposium on Information Theory.

[25] Duplinskiy A., Kiktenko E.O., Pozhar N.O., Anufriev M.N., Ermakov R., Kotov A., Brodskiy A., Yunusov R., Kurochkin V., Fedorov A.K. (2018). Quantum-secured data transmission in urban fiber-optics communication lines. J. Russ. Laser Res..

[26] Liu Z., Wu Z., Huang A. (2020). Blind information reconciliation with variable step sizes for quantum key distribution. Sci. Rep..

[27] Martinez-Mateo J., Pacher C., Peev M. (2015). Demystifying the Information Reconciliation Protocol Cascade. Quantum Inf. Comput..

[28] Walenta N., Burg A., Caselunghe D., Constantin J., Gisin N., Guinnard O., Houlmann R., Junod P., Korzh B., Kulesza N. (2014). A fast and versatile quantum key distribution system with hardware key distillation and wavelength multiplexing. New J. Phys..

[29] IEEE Computer Society LAN/MAN Standards Committee IEEE Standard for Information Technology-Telecommunications and Information Exchange between Systems-Local and Metropolitan Area Networks-Specific Requirements Part 11: Wireless LAN Medium Access Control (MAC) and Physical Layer (PHY) Specifications. *IEEE Std 802.11n-2009*. https://ieeexplore.ieee.org/document/5307322/.

[30] IEEE Computer Society and the IEEE Microwave Theory and Techniques Society LAN/MAN Standards Committee IEEE Standard for Local and Metropolitan Area Networks Part 16: Air Interface for Broadband Wireless Access Systems. *IEEE Std 802.16-2009*. https://ieeexplore.ieee.org/document/5062485/.

[31] Hu X.Y., Eleftheriou E., Arnold D.M. (2005). Regular and irregular progressive edge-growth tanner graphs. IEEE Trans. Inf. Theory.

